# Overdiagnosis among women attending a population-based mammography screening program

**DOI:** 10.1002/ijc.28052

**Published:** 2013-01-25

**Authors:** Ragnhild Sørum Falk, Solveig Hofvind, Per Skaane, Tor Haldorsen

**Affiliations:** 1 Department of Research, Cancer Registry of NorwayNorway; 2 Faculty of Health Sciences, Oslo and Akershus University College of Applied ScienceNorway; 3 Department of Radiology, Oslo University Hospital Ullevaal, University of OsloNorway

**Keywords:** cohort study, ductal carcinoma *in situ*, lead time, mammography screening, overdiagnosis

## Abstract

Increased incidence of ductal carcinoma *in situ* (DCIS) and invasive breast cancer (IBC) after introduction of organized screening has prompted debate about overdiagnosis. The aim was to examine the excess in incidence of DCIS and IBC during the screening period and the deficit after women left the program, and thereby to estimate the proportion of overdiagnosis. Women invited to the Norwegian Breast Cancer Screening Program were analyzed for DCIS or IBC during the period 1995–2009. Incidence rate ratios (IRRs) were calculated for attended *vs*. never attended women. The IRRs were adjusted by Mantel-Haenszel (MH) method and applied to a set of reference rates and a reference population to estimate the proportion of overdiagnosis during the women's lifespan after the age of 50 years. A total of 702,131 women were invited to the program. An excess of DCIS and IBC was observed among women who attended screening during the screening period; prevalently invited women aged 50–51 years had a MH IRR of 1.86 (95% CI 1.65–2.09) and subsequently invited women aged 52–69 years had a MH IRR of 1.56 (95% CI 1.45–1.68). In women aged 70–79 years, a deficit of 30% (MH IRR 0.70, 95% CI 0.62–0.80) was observed 1–10 years after they left the screening program. The estimated proportion of overdiagnosis varied from 10 to 20% depending on outcome and whether the women were invited or actually screened. The results highlight the need for individual data with longitudinal screening history and long-term follow-up as a basis for estimating overdiagnosis.

**What's new?**

Widespread screening for breast cancer means that more cancers are diagnosed than would be without the screening. Some of these cancers, however, would never have harmed the patient if left undetected. This overdiagnosis is difficult to measure, and in the current paper, the authors studied a cohort of women over a period of 10 years after they participated in cancer screening. They found an excess incidence of breast cancer during the initial screening period, followed by a deficit that lasted a decade after the women left the program. From this data, the authors estimated the proportion of overdiagnosis among women who participated in the screening program versus those who had not attended the screenings.

The incidence of ductal carcinoma *in situ* (DCIS) and invasive breast cancer (IBC) has increased substantially in recent decades, particularly in countries that offer mammographic screening.^1^–^4^ Consequently, the issue of overdiagnosis has been raised.^5^–^8^ Overdiagnosis can be defined as a histologically proven diagnosis of invasive or intraductal breast cancer that was detected at screening but that would not have surfaced clinically in the lifetime of the individual if no screening had been carried out.^9^,^10^ It is considered an epidemiological concept, since it is impossible to identify which tumors are overdiagnosed.

The primary purpose of screening is to reduce breast cancer-specific mortality by prevention or delay in the development of clinical disease through early detection. Lead time is the amount of time by which the diagnosis is advanced by screening. This shift leads to a temporary excess in incidence, which should be distinguished from overdiagnosis.^11^ Other factors influencing estimates of overdiagnosis are study method and estimation of incidence in the absence of screening,^8^ definition of denominators, periods of screening and length of follow-up.^12^

With the use of aggregated data, the effects of screening are difficult to identify.^13^ A more appropriate quantification of long-term effects of screening is enabled in a cohort of women where data on screening history are linked to incident cases (and deaths) from high-quality registries. In the present study, we used individual data from the Cancer Registry of Norway. We aimed to examine the extent of excess incidence of DCIS and IBC during the screening period and the deficit in a cohort of attending women after they had left the program. Based on these numbers, we estimated the proportion of overdiagnosis.

## Material and Methods

### Data sources

The Norwegian Breast Cancer Screening Program (NBCSP) is managed by the Cancer Registry of Norway. The program started in 1995/1996 in four pilot counties and expanded county by county from 1999 to 2005.^14^ Women aged 50–69 years are invited every second year to two-view mammography screening. The program is described in detail elsewhere.^14^

Information about invitations to and attendance of the NBCSP and about primary diagnosis of DCIS and IBC was available from the Cancer Registry of Norway. Reporting of invasive cancer (and certain preinvasive conditions) has been mandatory by law since 1953. Registration of breast cancer diagnoses is virtually 100%.^15^ Information about status (date of emigration/death) was available by linkage to the Central Population Registry and Cause of Death Registry at Statistics Norway using the unique personal identification number assigned to all inhabitants of Norway. Anonymous data on individual level were abstracted for our study.

### The study cohort

The study cohort included all women invited to the NBCSP, 1995–2009. Women diagnosed with DCIS or IBC before the first invitation date were excluded. The invitation date was defined as the postal date of the invitation, which was usually 3–4 weeks before the screening examination date. The women were followed up longitudinally from date of first invitation to date of diagnosis of DCIS or IBC, or censored at the date of emigration, death or study end (December 31, 2009), whichever occurred first.

The women's individual screening history was categorized according to their invitations and attendance. The women were at risk as prevalently invited from the first invitation date until the second invitation date, as subsequently invited from the second invitation date until 2 years after their last invitation received, and thereafter at risk as postinvited. Prevalent invitations were classified as either “attended” or “not attended.” Subsequent invitations were classified as “regularly” attended when the women had attended all invitation received so far, and as “never” attended when the women had not attended to any invitations so far. In the postperiod, the women were classified as either “ever” or “never” attending the program. Further, the postperiod was divided into 2-year periods since last attendance (or if “never” attended, since the last invitation), to enable exploring the pattern over time.

### Statistics

The incidence rate of DCIS and IBC was calculated as the number of primary cases of DCIS and IBC divided by the women-years at risk and presented as rates per 100,000 women-years (wys). Effects of attending the NBCSP were studied using incidence rate ratios (IRR) for attending *vs*. never-attending women. The data were stratified by county (counties with a common breast center were grouped together) and 1-year calendar periods, and the Mantel-Haenszel (MH) method was used to provide a pooled estimate of the IRRs across the strata. When all ages studied were combined, the IRRs were also stratified by 5-year age groups. The IRRs are presented crude and adjusted with 95% confidence interval (CI).

The NBCSP offers women 10 screening examinations during the age interval 50–69 years. However, the actual age at invitation varies because invitations are posted to predefined birth cohorts in the counties, and the length of the screening interval is 2 years. Thus, to cover the spread in age, the target age groups were extended by ±2 years in the estimation of the IRRs. To estimate the incidence for women who followed the official recommendations, a hypothetical cohort, we used the IRR for women aged 50–51 years invited prevalently and the IRRs for each of the 2-year intervals (52–53, …, 68–69 years) for women invited subsequently during the screening period. In the postperiod, we used the IRRs for each of the 2-year follow-up periods in women aged 70–79 years after they had left the program. This approach corresponds to the idea behind the expected age-specific incidence curve suggested by Boer *et al*.^16^ More lately, it has been applied on aggregated data to explore the influence of the three screening phases on IBC incidence.^17^,^18^

For quantifying the extent of overdiagnosis in a screening program, reference rates by age and a reference population are needed. We chose a life-table population according to mortality among Norwegian women in 2010, as given by Statistics Norway, as the reference population.^19^ Three sets of age-specific reference rates based on national data of IBC were considered to illustrate the effect of different shapes of the age-specific incidence rates. First, a modeling approach, we used the estimated relative rates by age from a former publication^20^ with the observed rates of women aged 40 years, 1993–1995, as the reference. Second, a period approach, we used observed rates from women diagnosed with IBC in 1980–1984. Third, a cohort approach, we used observed rates from a historical cohort of women born in 1903–1907. The three sets of reference rates were chosen to minimize the possible influence of use of hormone replacement therapy and use of unorganized screening on the age profile of the incidence. The reference rates were applied to the population and multiplied by the estimated MH IRRs to find the excess among attending women during the screening period and the deficit after screening ended. To form a single summary measure during the women's life, we summed the number of excess and deficit DCIS and IBC, and then divided it by the total number of cases diagnosed in women aged 50 years or older in the reference population to quantify the proportion of overdiagnosis. This denominator corresponds to method no. 2 in de Gelder *et al*.^12^ The CIs for the proportions of overdiagnosis were calculated using bootstrap methods. The individual data were resampled 1,000 times; then the IRR was calculated and applied for each of the three sets of references.

We calculated the extent of overdiagnosis for attending *vs*. never-attending women, whereas the majority of previous studies have provided intention-to-treat estimates of the effect of being invited to screening.^5^–^8^,^10^,^12^,^21^–^23^ To obtain comparable estimates, we multiplied our estimates of overdiagnosis with the compliance of the program under the assumption of equal baseline risk for attending and never-attending women. The compliance was calculated as the proportion of women who attended at least one of their received invitations during the study period. Not all studies include DCIS in their estimate of overdiagnosis,^5^,^8^,^10^,^24^ so to achieve more comparable estimates, we performed a secondary analysis using only primary cases of IBC as an endpoint.

A supplementary analysis was conducted to explore if there were differences in breast cancer risk between attending and nonattending women in the NBCSP (self-selection). We compared the incidence among women not yet invited and not attending by calculating MH IRRs in the implementation period of the program. The women-year at risk among those not yet invited to the program was computed using information on population statistics from Statistics Norway^19^ and from individual data among women invited to the NBCSP.

## Results

**Figure 1:**
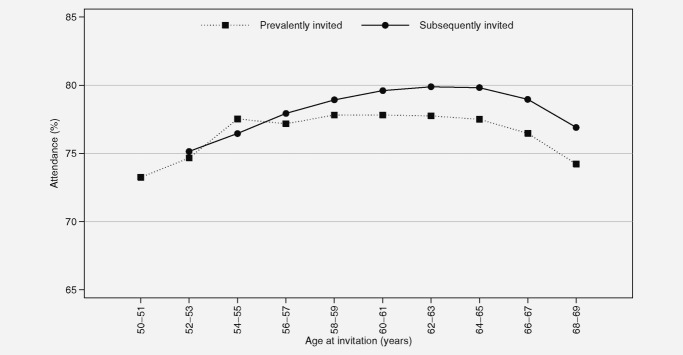
Attendance (%) by age for prevalently and subsequently invited women to the Norwegian Breast Cancer Screening Program.

We studied 702,131 women invited to the NBCSP during 1995–2009 who had no previous history of DCIS or IBC at the time of first invitation. The compliance to the program was 84%, while the attendance rate was 77% (based on 2,448,877 invitations). The attendance rate was higher for subsequently than prevalently invited women (Fig. 1). The median and maximum follow-up time was 7.0 and 14.2 years, respectively. In total, we observed 2,228 DCIS and 15,057 IBC during the study period.

The incidence of DCIS and IBC among prevalently invited women aged 50–51 years was 346/100,000 wys (attended 394/100,000 wys; not attended 211/100,000 wys). Among subsequently invited women aged 52–69 years, the incidence was 339/100,000 wys (regularly attended 357/100,000 wys; never attended 231/100,000 wys). During the 10-year postperiod, the incidence among women aged 70–79 years was 235/100,000 wys (ever attended 219/100,000 wys; never attended 318/100,000 wys) (Table 1). The incidence among never attended increased by age, but showed no trend by period.

**Table 1:** Number of cases, women-years and incidence rate ratios of DCIS and IBC among attending compared to never-attending women by screening phase and age

Target age (years)	Age in estimations (years)	Time in postperiod (years)	DCIS or IBC (*n*)	Women-years	DCIS or IBC (*n*)	Women-years	Crude IRR	MH IRR (95% CI)
Prevalently invited			Attended		Not attended			
50–51	48–53		1722	437,049	325	154,297	1.87	1.86 (1.65–2.09)
Subsequently invited			Regularly attended		Never attended			
52–53	50–55		1259	390,972	176	88,977	1.63	1.62 (1.38–1.90)
54–55	52–57		2065	658,114	271	137,143	1.59	1.61 (1.41–1.83)
56–57	54–59		2490	798,421	300	148,983	1.55	1.59 (1.40–1.79)
58–59	56–61		2658	780,136	283	126,576	1.52	1.59 (1.40–1.80)
60–61	58–63		2693	739,974	284	109,325	1.40	1.46 (1.29–1.65)
62–63	60–65		2631	681,743	258	95,588	1.43	1.48 (1.30–1.68)
64–65	62–67		2457	613,251	224	84,746	1.52	1.55 (1.35–1.78)
66–67	64–69		2288	550,406	197	77,623	1.64	1.69 (1.46–1.96)
68–69	66–71		1670	443,450	164	65,570	1.51	1.58 (1.34–1.85)
52–69	50–71		7306	2,046,485	788	340,499	1.54	1.56 (1.45–1.68)
Post–period			Ever attended		Never attended			
70–71	68–73	1–2	281	161,384	93	33,491	0.63	0.60 (0.47–0.77)
72–73	70–75	3–4	306	129,041	84	24,421	0.69	0.71 (0.55–0.91)
74–75	72–77	5–6	204	82,444	41	14,101	0.85	0.92 (0.66–1.29)
76–77	74–79	7–8	104	42,796	20	6744	0.82	0.85 (0.52–1.39)
78–79	76–81	9–10	47	18,258	9	3214	0.92	0.88 (0.42–1.83)
70–79	68–81	1–10	1089	497,508	317	99,761	0.69	0.70 (0.62–0.80)

Abbreviations: DCIS: ductal carcinoma *in situ*; IBC: invasive breast cancer; IRR: incidence rate ratio of attending *vs*. never-attending women; MH: Mantel-Haenszel method, used to adjust for county, calendar period (and age when considering all ages in combined); CI: confidence interval.

The IRRs between attended and never attended are presented in Table 1, separately for the three screening phases. In prevalently invited, attending women had an increased risk of DCIS and IBC (MH IRR 1.86, 95% CI 1.65–2.09) compared to women who did not attend. In subsequently invited, women with regular attendance had an increased risk (MH IRR 1.56, 95% CI 1.45–1.68) compared to women who never attended. The MH IRRs showed no pattern by age.

In the postperiod, a significant deficit of 30% was observed for women who ever *vs*. never attended (MH IRR 0.70, 95% CI 0.62–0.80). The deficit was greatest in the first years after the women left the program. In the latest three 2-year periods a deficit was still observed, though nonsignificant. Seventy percent of the deficit during the 10-year period was observed within 5 years. As the NBCSP was gradually implemented, the counties have different follow-up times. The four pilot counties (40% of the population) with the longest follow-up time had a smaller deficit during the first 6 years in the postperiod (MH IRR 0.77, 95% CI 0.63–0.94) than the nonpilot counties (MH IRR 0.61, 95% CI 0.49–0.76), which have a shorter follow-up time for ever- *vs*. never-attending women.

**Figure 2:**
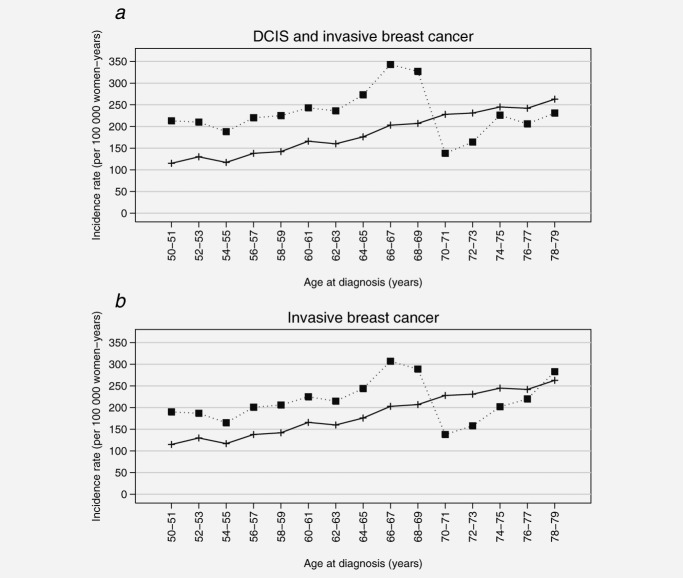
Incidence of DCIS and IBC (*a*) and IBC (*b*) per 100,000 women-years by age at diagnosis. Solid line: reference (period approach). Dotted line: reference multiplied with the MH IRRs. Abbreviations: DCIS: ductal carcinoma *in situ*; IBC: invasive breast cancer; MH IRR: incidence rate ratio of attending *vs*. never-attending women adjusted by county and calendar period using the Mantel-Haenszel method.

In the hypothetical cohort, overdiagnosis of DCIS and IBC among women attending 10 invitations compared to women who never attended was estimated at 17–20% for the set of references rates (Table 2). For IBC alone, the estimated proportions of overdiagnosis were 11–13%. The excess and deficit during the affected age span are illustrated graphically in Figure 2. The estimated proportions of overdiagnosis among women invited (intention to treat) were 14–17% for DCIS and IBC, and 10–11% for IBC only (Table 2). Details in the calculation of overdiagnosis are shown in Table 3.

**Table 2:** Estimated proportion of overdiagnosis among attending and invited women after implementation of the screening program

	Attended^1^		Invited^2^	
	
Reference	OD (%)	95% CI	OD (%)	95% CI
DCIS and invasive breast cancer				
Modeling approach	19.6	12.1–27.1	16.5	10.2–22.7
Period approach	19.4	11.8–27.0	16.3	9.9–22.7
Cohort approach	16.5	9.1–23.9	13.9	7.6–20.1
Invasive breast cancer				
Modeling approach	13.4	4.7–22.1	11.3	3.9–18.6
Period approach	13.3	4.0–22.6	11.2	3.3–19.0
Cohort approach	11.4	2.7–20.1	9.6	2.2–16.9

1 Women who attended screening compared with women who never attended.

2 Calculated as the estimate among attending women multiplied by the compliance of the program (84%).

Abbreviations: DCIS: ductal carcinoma in situ; OD: estimated proportion of overdiagnosis; CI: confidence interval.

**Table 3:** Details in calculation of estimated proportion of overdiagnosis

Age (years)	Population^1^	MH IRR^2^	Reference incidence^3^	Expected^4^	Excess/deficit
50	31,586	1.86	114.9	36.3	31.1
51	31,520			36.3	31.1
52	31,446	1.62	129.9	40.8	25.3
53	31,367			40.7	25.2
54	31,279	1.61	117.0	36.6	22.1
55	31,195			36.5	22.1
56	31,099	1.59	138.5	43.1	25.3
57	30,999			42.9	25.2
58	30,882	1.59	141.5	43.7	25.7
59	30,746			43.5	25.5
60	30,614	1.46	166.4	50.9	23.3
61	30,458			50.7	23.2
62	30,296	1.48	159.9	48.4	23.2
63	30,109			48.1	23.0
64	29,922	1.55	176.1	52.7	28.9
65	29,703			52.3	28.7
66	29,438	1.69	202.8	59.7	41.3
67	29,197			59.2	40.9
68	28,912	1.58	207.5	60.0	34.5
69	28,607			59.4	34.1
70	28,317	0.60	228.5	64.7	−25.6
71	27,979			63.9	−25.3
72	27,616	0.71	230.8	63.7	−18.4
73	27,195			62.8	−18.1
74	26,691	0.92	245.2	65.4	−5.0
75	26,193			64.2	−4.9
76	25,620	0.85	242.0	62.0	−9.1
77	22,993			60.5	−8.9
78	24,353	0.88	263.5	64.2	−8.0
79	23,653			62.3	−7.8
80–99	227,723	1.00	277.7	632.5	0
Sum				2,208.1	428.4^5^

1 Decrement of 31,586 women (observed number of women 50 years old in Norway in 2010) according to the observed mortality in 2010 as given by Statistics Norway.

2 MH IRR of ductal carcinoma *in situ* and invasive breast cancer as given in Table 1.

3 Observed incidence of invasive breast cancer in 1980–1984 per 100,000 women-years (Period approach).

4 Expected number of cases (ductal carcinoma *in situ* and invasive breast cancer).

5 Estimated proportion of overdiagnosis: 428.4/2,208.1 = 19.4%.

Abbreviation: MH IRR: incidence rate ratio of attending vs. never-attending women adjusted for county and calendar period using the method of Mantel-Haenszel.

In exploring possible self-selection bias, we found no statistical differences in the IRRs (MH IRR 0.94, 95% CI 0.83–1.07) in women younger than 55 years, whereas older women who did not attend (291/100,000 wys) had a higher incidence of DCIS and IBC than those who were not invited (239/100,000 wys).

## Discussion

In this nationwide study based on individual data, we followed up the women longitudinally from the date of first invitation to explore the incidence of DCIS and IBC during the screening period and 10 years after they left the organized program. An excess of cases was observed throughout the screening period followed by a deficit that still exists 10 years after the women left the screening program.

The European guidelines state that the acceptable detection rate for breast cancer (DCIS and IBC) should be 3 and 1.5 times the background incidence for women screened initially (e.g. aged 50–51 years) and in subsequent regularly screened, respectively.^25^ Because we reported incidence rates, the threshold values had to be converted. Assuming that interval cancer incidence is 40% of background incidence (an acceptable level according to the guidelines) each year in the 2-year screening period. Then three times the background incidence plus two times 40% of background incidence adds up to 3.8 times of background incidence in 2 years, which gives a yearly average incidence of 1.9 in prevalently screened women. Correspondingly, for subsequently screened women, at least 1.15 [(1.5 + 2 × 0.4)/2] is acceptable according to the guidelines. We observed an IRR in line with the lower limit of the guidelines among prevalently invited (1.86) and an IRR above the lower limit in subsequently invited women (1.56). The guidelines do not indicate any level of relative incidence after screening ends. Compared to IRRs of IBC among screened women presented from aggregated data,^18^ we observed higher ratios in the screening period and lower in the postperiod.

When overdiagnosis is quantified, the incidence should ideally be compared in a cohort of attending and not-attending women enrolled in a randomized controlled trial with lifelong follow-up.^8^ An excess of cases at the end of follow-up in the group of attending women would reflect the magnitude of overdiagnosis. Estimates of overdiagnosis in population-based screening programs must control for the effect of lead time. Two methods have been suggested, either adjustment with statistical methods or adequate follow-up that allows for the subsequent deficit.^16^,^26^ For this method of compensatory drop, Biesheuvel *et al*. suggested that at least 5 years of follow-up was needed; Paci *et al*. suggested that 90% of incremental cases are expected to be decremented within 5 years after screening.^8^,^21^ On the basis of our observed deficit throughout the 10-year period, we suggest that more than 10 years of follow-up is needed. Furthermore, 70% of the deficit in the 10-year postperiod was observed during the first 5 years. The proportion of overdiagnosis might be overestimated because the possible deficit after 10 years is not incorporated. Furthermore, in analysis by county, the nonpilot counties had larger deficits during the first 6 years in the postperiod than the pilot counties. If this deficit persists, it indicates a lower estimate of overdiagnosis when all counties complete more than 10 years of follow-up. This suggests that the lead time of DCIS and IBC is longer than previously stated.^7^

Studies have shown large variation in the estimates of overdiagnosis, ranging from less than 1 to 54%.^8^ Estimates have been higher in studies based on aggregated data^5^,^6^,^24^ than in those based on individual data.^21^–^23^,^27^,^28^ This applied both for studies with and without DCIS included, and in studies related to invited or attending women. The most comparable study to ours was performed by Puliti *et al*., who also conducted an observational cohort study comparing attendees and nonattendees. They reported 10–15% overdiagnosis of DCIS and IBC among women screened aged 50–69 years, which they suggest was possibly underestimated.^27^

Previous attempts have been made to estimate overdiagnosis with Norwegian data. Based on aggregated data, authors reported 15–25,^5^ 37,^6^ and 54%^24^ overdiagnosis of IBC among women invited to the NBCSP. Our result based on individual data was 10–11%. The difference may be due to several factors. Their analysis was based aggregated data on groups of women that approximate the target population of the NBCSP without using individual information about actual invitation to the program (or attendance). For example in the postperiod in which a compensatory drop should be enabled, they included never-invited women.^29^,^30^ Further, their studies had shorter follow-up, and they may have intended to estimate overdiagnosis within the observation period, while we have estimated overdiagnosis in a hypothetical cohort within lifetime.

Studies including DCIS are expected to yield higher estimates of overdiagnosis than those limited to IBC because including DCIS implies earlier diagnosis and greater possibility of death from other causes than breast cancer before diagnosis without screening. Furthermore, it is suggested that some DCIS does not progress.^31^ The lifetime risk of progression from DCIS to IBC is unknown. Nevertheless, in epidemiological studies both increased risk of subsequent malignancy and mortality have been reported;^32^ even low-grade DCIS has been associated with a cancer rate of 39%, of which 45% of the women died from metastatic disease after 30 years of follow-up.^33^ Thus, the conundrum in overdiagnosis is that clinicians never know at the time of diagnosis which tumor is overdiagnosed, and consequently overtreatment is inevitable.

With access to individual data, both intention-to-treat estimates and results for those actually attending the program might be presented. In studies comparing attendees and nonattendees, the possible bias of self-selection has to be considered. Our results indicate no bias for women below 55 years. Among women 55 years and older we observed a higher incidence of DCIS and IBC in not-attending compared to not-invited women. This may indicate that attendee women had used mammography to a larger extent before being invited to the program than nonattendees, or that attendees and nonattendees have different baseline risk for DCIS and IBC. However, in the literature there is no consistent evidence about different underlying incidence risks in attendees *vs*. nonattendees.^27^ The question of self-selection represents a source of uncertainty for our estimates.

Reliable estimation of IRRs depends on equal distribution of strong risk factors for breast cancer among attending and never-attending women within strata. We adjusted for the presence of potential confounders where the greater part of the variation is described by age, calendar period and county (e.g. use of hormone therapy^20^). The modest differences between the crude and adjusted IRRs indicated limited confounding through age, period and county. Further, our choice of reference rates had moderate influence on the estimates of overdiagnosis.

Lack of individual information on use of mammography outside the program might bias our results. For example if some women continue to undergo screening after the upper age limit of the NBCSP, the expected drop will be delayed, and preclude the estimate. A review of mammographic activity in Norway showed that for women aged over 69, up to 9% had mammography in 2005.^34^ Introduction of digital mammography is another factor that may influence the results. In the NBCSP, examinations performed with digital mammography increased from 1% in 2000 to 31% in 2007.^2^ Use of digital mammography has shown higher detection rates of both DCIS and IBC,^35^ and estimates of overdiagnosis are expected to increase.^36^

Estimation of overdiagnosis depends on observations in women after end of screening. Since service programs have existed for only a few decades, limited follow-up after end of screening are available. Our estimates are hampered by lack of data on women who have been screened during the greater part of the screening range (50–69 years) and limited observations after the end of screening for screened women (70 years and older). Actually, the women in the birth cohort 1927–1928 in the pilot counties had the longest follow-up after screening, 10 years, but these women were invited only twice. This limits possibilities to study deficit as a function of screening history. Thus, we considered ever attended instead of regularly screened women in the postperiod to have sufficient women-years.

In conclusion, we observed excess incidence of DCIS and IBC in the screening period and a deficit that prevailed 10 years after the women left the program. Individual data with longitudinal screening history and long-term follow-up after end of screening are necessary for valid estimates of overdiagnosis.

## Abbreviations

CI: confidence interval
DCIS: ductal carcinoma in situ
IBC: invasive breast cancer
IRR: incidence rate ratios
MH: Mantel-Haenszel
NBCSP: Norwegian Breast Cancer Screening Program: wys: women-years
